# Design of an elastin-layered dermal regeneration template^☆^

**DOI:** 10.1016/j.actbio.2016.11.054

**Published:** 2017-04-01

**Authors:** Suzanne M. Mithieux, Anthony S. Weiss

**Affiliations:** a Charles Perkins Centre, University of Sydney, NSW 2006, Australia; b School of Life and Environmental Sciences, University of Sydney, NSW 2006, Australia; c Bosch Institute, University of Sydney, NSW 2006, Australia

**Keywords:** Elastin, Elastic fiber, Tropoelastin, Deep dermis, Integra

## Abstract

We demonstrate a novel approach for the production of tunable quantities of elastic fibers. We also show that exogenous tropoelastin is rate-limiting for elastin synthesis regardless of the age of the dermal fibroblast donor. Additionally, we provide a strategy to further enhance synthesis by older cells through the application of conditioned media. We show that this approach delivers an elastin layer on one side of the leading dermal repair template for contact with the deep dermis in order to deliver prefabricated elastic fibers to a physiologically appropriate site during subsequent surgery. This system is attractive because it provides for the first time a viable path for sufficient, histologically detectable levels of patient elastin into full-thickness wound sites that have until now lacked this elastic underlayer.

**Statement of Significance:**

The scars of full thickness wounds typically lack elasticity. Elastin is essential for skin elasticity and is enriched in the deep dermis.

This paper is significant because it shows that: (1) we can generate elastic fibers in tunable quantities, (2) tropoelastin is the rate-limiting component in elastin synthesis *in vitro*, (3) we can generate elastin fibers regardless of donor age, (4) we describe a novel approach to further increase the numbers and thickness of elastic fibers for older donors, (5) we improve on Integra Dermal Regeneration Template and generate a new hybrid biomaterial intended to subsequently surgically deliver these elastic fibers, (6) the elastic fiber layer is presented on the side of Integra that is intended for delivery into its physiologically appropriate site i.e. the deep dermis.

## Graphical abstract

**Figure:**
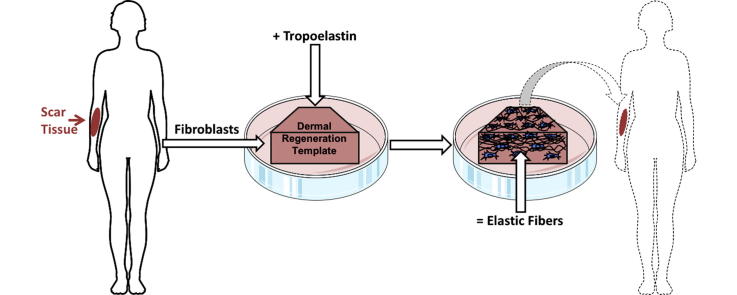

## 1 Introduction

Elastin is integral to the extracellular matrix of vertebrate tissues such as blood vessels, lungs and skin, where it provides the structural integrity and elasticity required for mechanical stretching of these tissues during normal function [1]. Elastin’s three-dimensional architecture reflect its physical environment and biological demands: elastic vessels carry blood in the vasculature, the lung expands and contracts with each breath, and fibers in the dermis facilitate skin stretching and recoil. In the dermis, elastin is arrayed in the form of fibers, the dominant component of which is the elastin polymer. Although elastin is one of the most durable human proteins lasting as long as the human host [2], [3] dogma states that elastic fiber synthesis in tissues including the dermis effectively ceases in early childhood [4]. After this, the regeneration of elastic fibers in full thickness wounds is severely compromised [5]. There is a strong demand for *de novo* elastic fiber synthesis, particularly in the deep dermis, in order to maintain viable elasticity and skin function. Elastin is mainly present in the reticular portion of the dermis where large diameter elastic fibers sit deep within the tissue and are parallel to the skin surface [6].

Although dermal fibroblasts are able to secrete elastin, its synthesis is repressed in the skin and many adult tissues by post-transcriptional mechanisms [7], [8]. Therefore there is an ongoing search for mechanisms that can quantitatively deliver elastic fibers into a patient’s deep dermis such as when full thickness dermal substitute products like Integra Dermal Regeneration Template (IDRT) are used to repair full thickness wounds. Indeed, in the use of materials such as IDRT, the regeneration of the elastic fiber system is acknowledged as integral to the functional performance and development of the next generation of dermal substitutes [9].

We reasoned that we can address this demand for an improved dermal substitute material by encouraging patient fibroblasts to synthesize significant quantities of elastic fibers. This harmonizes with the use of cells because they provide attractive treatments for dermal wounds and scars where they are increasingly acknowledged as an important part of viable tissue repair [10], [11].

Elastin is predominantly made of the one protein tropoelastin which is assembled with the assistance of an ensemble of elastic fiber proteins [12]. Elastin formation occurs in a stepwise process involving tropoelastin association, massive molecular deposition and cross-linking [13]. Microfibrillar proteins like members of the fibrillin and fibulin families are needed for elastic fiber organization *in vivo* [14], [15], [16], [17], [18]. Recently, we and others have found that, *in vitro*, tropoelastin contains multiples signals that dictate precise spatial and temporal elastin assembly [19], [20], [21], [22], [23], [24]. Although other elastic fiber proteins are important, elastin is over nine times more abundant than microfibrillar components, and therefore tropoelastin-tropoelastin interactions are significant [25]. Here, we reason that the rate limiting step in elastin formation might be the supply of tropoelastin. We explore this concept and show that, using fibroblasts from human donors ranging in age from neonatal to 92 years old, *de novo* elastic fibers can be deposited into the extracellular matrix when tropoelastin is supplied exogenously. Furthermore, we investigate ways in which elastogenesis can be enhanced in this system and use these discoveries to enrich an IDRT full-thickness dermal substitute by endowing it with a network of elastic fibers that is positioned appropriately for delivery into the deep dermis.

Surgery and tissue repair can restore function if components are available. A long-sought after goal in elastic tissue engineering has been to restore elastic fibers to tissues such as the skin. We present a novel strategy towards the use of patient-donated skin fibroblasts to deliver tailored quantities of elastic fibers to this location. This technology lays the foundation for the repair of full-thickness wounds and the contemporaneous delivery of a dense net of elastic fibers deep within the human dermis.

## 2 Materials and methods

### 2.1 Human dermal fibroblasts

Human dermal fibroblasts used in this study were sourced from neonatal males (NHF45C ThermoFisher; NHF8909 gift of X. Q. Wang, University of Queensland, Australia), a 10 year old male (GM03348 Coriell Institute for Medical Research), a 31 year old male (obtained from a consenting burns patient in the Burns Unit at Concord Repatriation General Hospital, NSW, Australia in accordance with the approval of the Hospital Research and Ethics Committee), a 51 year old male (142BR Sigma) and a 92 year old male (AG04064 Coriell Institute for Medical Research).

### 2.2 Tropoelastin

Recombinant human tropoelastin isoform SHELΔ26A (synthetic human elastin without domain 26A) corresponding to amino acid residues 27–724 of GenBank entry AAC98394 (gi 182020) was purified from bacterial culture as described previously [26], [27] (Elastagen Pty Ltd).

### 2.3 Cell culture

#### 2.3.1 Elastogenesis model

Human dermal fibroblasts (5 × 104 cells) were seeded on glass cover slips in the wells of 12 well tissue culture plates in Fresh Media (FM) containing DMEM (Life Technologies) with 10% (v/v) fetal bovine serum (FBS; Life Technologies) and 1% (v/v) Pen/Strep (Sigma). Cells were cultured at 37 °C 5% CO_2_ and the media was changed every 2–3 days. On Day 10 of culture 1 mg tropoelastin (filter sterilized; 10 mg/ml in phosphate buffered saline (PBS)) was added to each well and the cells were cultured for a further seven days, with media changes on days 13 and 15. Control cell samples with no tropoelastin addition were cultured for 17 days. At 1, 3 or 7 days post-tropoelastin addition the cultured cells were washed twice in PBS then fixed with 4% (w/v) paraformaldehyde for 20 min and quenched with 0.2 M glycine. The cells were incubated with 0.2% (v/v) Triton X-100 for 6 min, blocked with 5% (w/v) bovine serum albumin at 4 °C overnight, and stained with a 1:500 dilution of BA4 mouse anti-elastin antibody (Sigma) for 1.5 h and a 1:100 dilution of anti-mouse IgG-FITC antibody (Sigma) for 1 h. The coverslips were mounted onto glass slides with ProLong Gold anti-fade reagent with DAPI (Invitrogen). Slides were left to set for 24 h then analyzed using a confocal microscope.

#### 2.3.2 Conditioned media

Conditioned media (CM) was prepared by collecting media from 3 day cultures of neonatal dermal fibroblasts in FM, filter sterilizing and mixing in a 1:1 ratio with DMEM containing 20% (v/v) FBS and 1% (v/v) Pen/Strep. Medium containing 20% FBS was added to account for serum components that had been depleted from the media collected from the 3 day FM cultures of neonatal fibroblasts. The final FBS concentration in the CM was up to 15%. To control for this possibility a medium containing DMEM with 15% (v/v) FBS and 1% (v/v) Pen/Strep was also tested. Fibroblasts sourced from a 51 year old male (142BR) were cultured in FM, CM or control media for 17 days with 1 mg tropoelastin (filter sterilized; 10 mg/ml in PBS) added on Day 10. Samples were fixed and stained as described above.

For size fractionation experiments CM was spun through Amicon Ultra-15 Centrifugal Filter Units (Millipore;100 kDa and 30 kDa MWCO). Concentrated solutions of >100 kDa and 30–100 kDa were rediluted in DMEM with 10% (v/v) FBS and cells were cultured in each media as described above.

#### 2.3.3 RNA extraction

Triplicate samples of fibroblasts (1 × 105 cells) were seeded into the wells of 6 well tissue culture plates and cultured for 11 days in FM (Neonatal and 142BR) or CM (142BR) with media changes every 2–3 days. Cells were harvested and RNA extracted using an RNeasy Mini Kit (Qiagen).

#### 2.3.4 Repeated tropoelastin supplementation

Human dermal fibroblasts were cultured for 31 days in FM as described above. On days 10, 17 and 24 tropoelastin (1 mg filter sterilized; 10 mg/ml in PBS) was added to the wells such that the cultures were supplemented with 1, 2 or 3 additions of tropoelastin. Non-supplemented cells were also cultured. Samples were fixed and stained as described above.

#### 2.3.5 Preparation of dermal substitute containing patient cells and elastic fibers

IDRT (Integra Life Sciences Corporation, Plainsboro, NJ; 1.5 × 1.5 cm) squares were placed in the wells of 12 well cell culture plates and seeded with neonatal human dermal fibroblasts (2 × 105 cells in 200 μl FM). After 1 h at 37 °C 5% CO_2_ a further 3 ml of FM was added to each well. Cells were cultured on IDRT for up to 33 days with media changes every 2–3 days. At days 12, 19 and 26 tropoelastin (1 mg filter sterilized; 10 mg/ml in PBS) was added to the wells. At days 19, 26 and 33 samples were fixed and stained following 1, 2 or 3 additions of tropoelastin. IDRT samples cultured for 33 days with cells and no tropoelastin supplementation or with no cells and 3 additions of tropoelastin were also prepared. Samples were fixed in 10% formalin. For cross-section imaging samples were embedded in paraffin, sectioned and stained with either hematoxylin and eosin or BA4 mouse anti-elastin antibody and an HRP conjugated anti-mouse secondary antibody (Dako Envision system HRP labelled polymer anti-mouse) and visualized using Liquid DAB + substrate chromogen system (Dako). A surface view was obtained using confocal microscopy of samples stained as described above.

### 2.4 RNA analysis

For each condition, triplicate samples of RNA were probed and analyzed by microarray analysis using Affymetrix Human Prime View (U219) array at The Ramaciotti Centre for Gene Function Analysis NSW Australia. Expression Console 1.0 software (Affymetrix) was used to normalize data using RMA-sketch, which were then annotated using HuGene 1.0 ST v1 library and annotation files. Signal intensities were averaged between triplicates and SD was determined. For detection of differentially expressed genes, a *p*-value less than 0.05 was used in combination with a fold-change cut-off above 2.0 and signal intensity above background (i.e., 200) level. Where multiple probe sets for the same gene showed differential expression, the probe set with the largest signal intensity is reported as representative.

### 2.5 Confocal microscopy

Fluorescently immunostained samples were visualized with an Olympus FluoView FV1000 confocal microscope using laser excitation at 405 nm to detect DAPI fluorescence, 488 nm to detect FITC fluorescence and 559 nm to detect elastin autofluorescence. Images were analyzed using ImageJ software [28]. Z-stacks were taken from 10 fields of view (FOV) per sample, converted to maximum projection images and analyzed for total area of elastic fibers and relative fiber numbers. In all cases results from 10 FOV were averaged to give a result per sample. For percent area of tropoelastin staining analysis the automated, software-generated threshold was used to exclude background pixels on each image. The number of green pixels was measured and converted to % per total area. To compare relative fiber numbers, three parallel lines were drawn and evenly distributed across each FOV. The number of fibers crossing each line was counted, added together and divided by three. The number of cell nuclei per FOV was also counted.

### 2.6 Statistics

Student’s unpaired t tests (RNA analysis, relative fiber number analysis) or one-way ANOVA with Bonferroni multiple comparison tests (all other analyses) were performed using Graph Pad Prism version 6.07 software. Statistical significance was accepted at values of *p* < 0.05. Data are presented as mean ± SEM for CM and multiple tropoelastin addition experiments and mean ± SD for RNA analysis. In the figures, significance is indicated by asterisks (^*^p < 0.05, ^**^p < 0.01, ^***^p < 0.001, ^****^p < 0.0001).

## 3 Results and discussion

### 3.1 Elastogenesis by human dermal fibroblasts

**Fig. 1:**
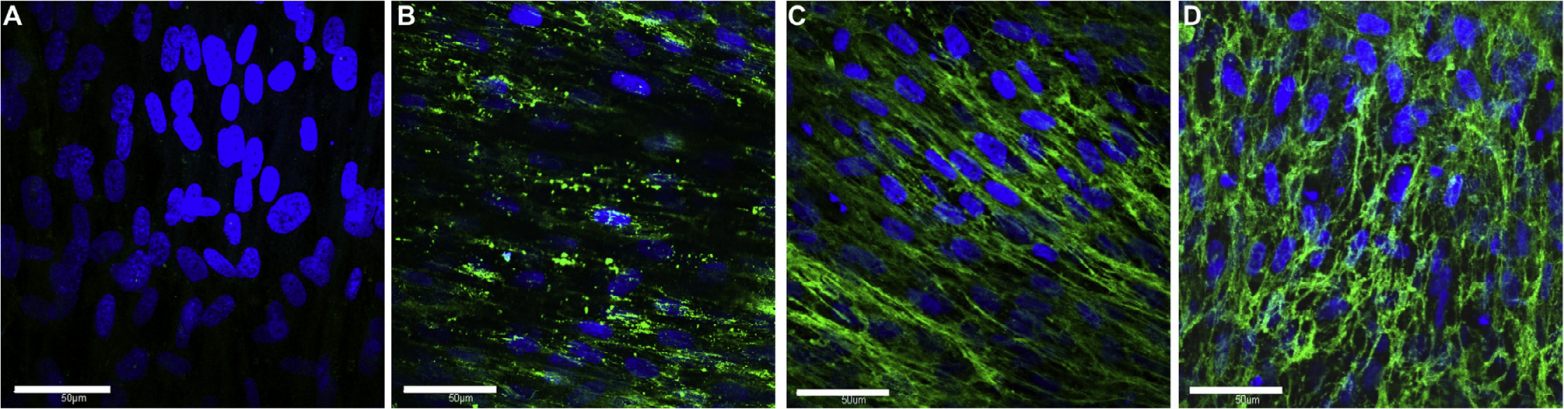
Model *in vitro* elastogenesis system. Elastic fiber formation by human neonatal dermal fibroblasts in the absence of exogenous tropoelastin (A) or 1(B), 3(C) and 7(D) days post tropoelastin addition. Elastin fibers (green) were stained with BA4 anti-elastin antibody and anti-mouse FITC conjugated secondary antibody. Nuclei (blue) were stained with DAPI. Images were obtained on an Olympus FluoView FV1000 confocal microscope. Scale bar = 50 μm. (For interpretation of the references to colour in this figure legend, the reader is referred to the web version of this article.)

**Fig. 2:**
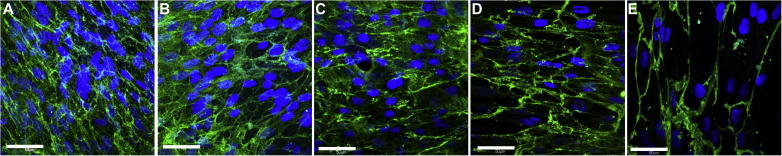
Elastogenesis at different ages. Comparison of elastin networks formed 7 days after tropoelastin addition to cultured dermal fibroblasts sourced from different age groups –neonatal (A), 10 (B), 31 (C), 51 (D) and 92 (E) years old. Confocal microscopy images were taken of cultures stained for elastin (green) and nuclei (blue). Scale bar = 50 μm. (For interpretation of the references to colour in this figure legend, the reader is referred to the web version of this article.)

We and others [20], [29], [30], [31] have used *in vitro* cell culture models with the addition of recombinant tropoelastin to investigate elastogenesis by cells. In our model system, human dermal fibroblasts are cultured for 10–12 days prior to the addition of purified recombinant human tropoelastin and then cultured for up to a further 7 days. In the absence of exogenous tropoelastin no elastin fiber synthesis is evident (Fig 1A). Following tropoelastin addition, the protein is deposited into the extracellular matrix as globules (Fig 1B) as is also seen during normal elastogenesis [32], [33]. Subsequent fiber formation is initially aligned in the direction of cells (Fig 1C) before an extensively branched elastic fiber network is generated (Fig 1D). Using this model, we found that human dermal fibroblasts sourced from a wide range of donor ages (neonatal, 10, 31, 51 and 92 years old) can make elastic fibers when they are supplied with tropoelastin (Fig 2). However the fiber architecture changes with age; cells sourced from older age groups produce fewer, thicker and less branched fibers. This indicates that dermal treatments requiring repair or replacement of damaged elastic fiber networks in older individuals may be less effective.

### 3.2 Enhancing elastogenesis with CM

**Fig. 3:**
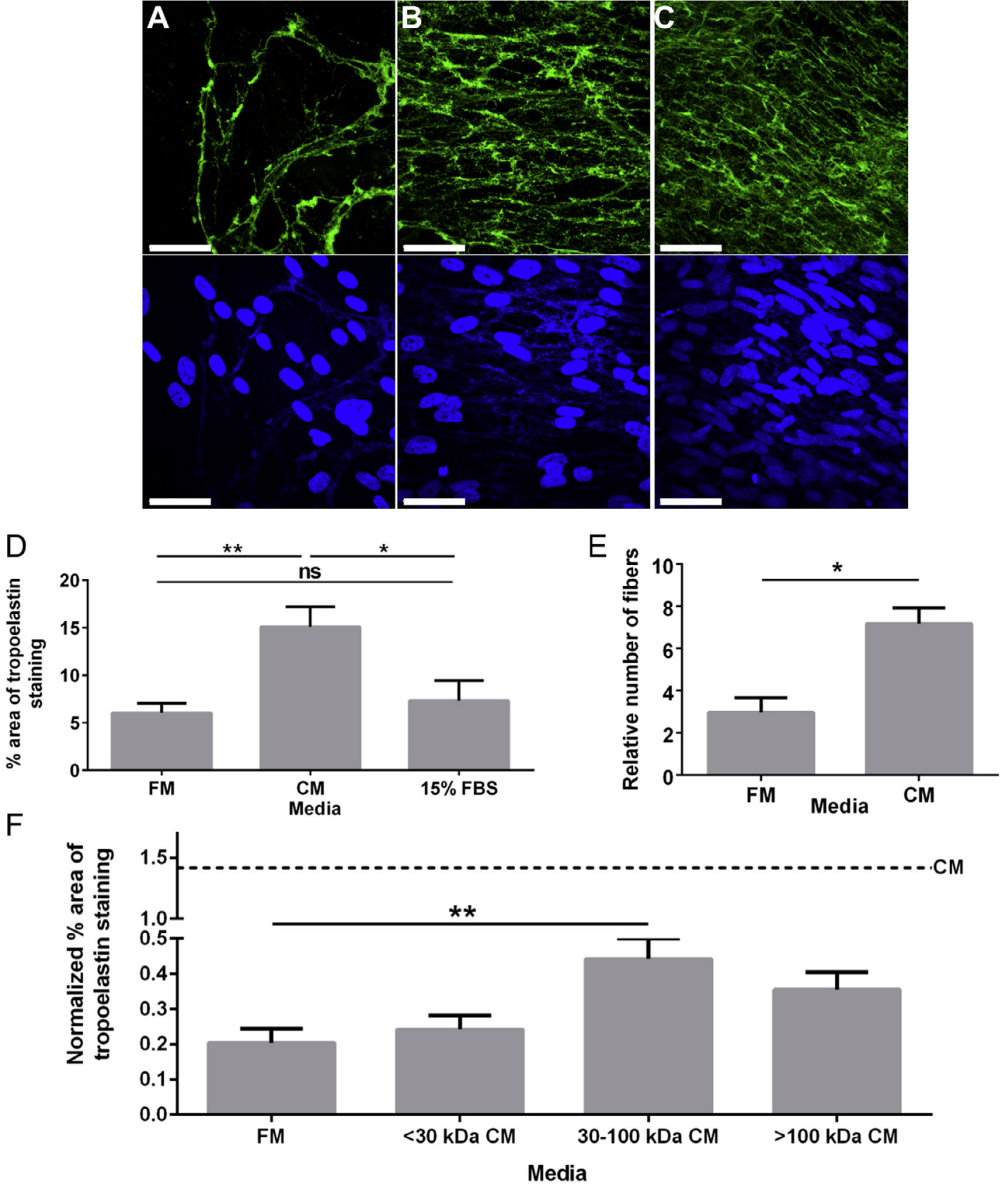
Enhanced elastogenesis through use of CM. Elastin fiber formation by dermal fibroblasts, sourced from either a 51 year old (A and B) or a neonatal (C) and cultured in FM (A and C) or CM (B) for 17 days. Tropoelastin was added on Day 10. Confocal microscopy images were taken of cultures stained for elastin (green; upper panel) and nuclei (blue; lower panel). Scale bar = 50 μm. Image analysis of 10 fields of view per experiment demonstrated the enhancing effect of CM media on tropoelastin deposition (D; n = 6) and fiber numbers (E; n = 3) on cells sourced from a 51 year old and cultured for 17 days with tropoelastin addition on Day 10. Image analysis of the same cells grown in CM that had been divided based on the molecular weight range (<30 kDa, 30–100 kDa and >100 kDa) of its components (F). Ten fields of view were analyzed per culture medium and normalized using the average number of nuclei seen in that medium. ^*^p < 0.05; ^**^p < 0.01. (For interpretation of the references to colour in this figure legend, the reader is referred to the web version of this article.)

Given the ability of neonatal cells to produce extensive elastic fiber networks, we explored the effect of neonatal dermal fibroblast CM on elastogenesis. Fibroblasts were sourced from a 51-year old and treated with neonatal CM. Tropoelastin was then added to initiate elastogenesis. Compared to growth in FM (Fig 3A), CM (Fig 3B) resulted in a 2.5-fold increase in tropoelastin deposition into the extracellular matrix (Fig 3D) which was accounted for by an associated 2.5-fold increase in the numbers of elastic fibers (Fig 3E). No difference in tropoelastin deposition was seen when FM was compared with control medium (15% FBS; Fig 3D). With the addition of CM, these older cells showed elastin networks that were comparable to those of neonatal cells (Fig 3C). In all cases the number of nuclei per field of view was indistinguishable regardless of whether the older cells were grown in FM or CM.

Microarray analyses on triplicate samples of fibroblasts cultured for 11 days in FM (neonatal and 51 years old) or CM (51 years old) were performed to investigate the mechanism by which CM enhanced elastogenesis in older cells. Cells sourced from the 51 year old showed comparable (within 2-fold) levels of gene expression irrespective of whether they were in CM or FM, and confirmed that there was no significant change in tropoelastin expression (signal intensities 1746 ± 228 (CM), 2060 ± 144 (FM); *p* = 0.113). These findings support a model where soluble factors in CM have a direct influence on the development of the elastic matrix by the older cells, rather than on gene expression. On this basis, we compared expression data from neonatal cells to older cells where both were grown in FM. Given that older cells are capable of making elastic fibers, the resulting data were filtered to only include extracellular matrix-associated proteins that were expressed by both neonatal and older cells, with a signal intensity > 200, and showed statistically significant (*p* < 0.05) increased expression levels (>2-fold) by the neonatal cells. This resulted in the identification of 7 differentially expressed genes (Table 1).

The majority of the identified targets (fibrillin 2, fibulin 1, microfibrillar associated protein 4 and latent TGFβ binding protein 1) are known elastic fiber components. Fibrillin-2 (315 kDa) predominantly regulates the early process of elastic fiber assembly [34]. It is expressed during early development with expression turned off shortly after birth. During fetal expression fibrillin 2 contributes to the microfibrillar core structure which is then overlaid postnatally by fibrillin 1 [35]. Fibulin 1 (70–100 kDa) binds tropoelastin [36], [37]. Microfibrillar associated protein 4 (MFAP4; 36 kDa monomer) binds tropoelastin, desmosine, fibrillin 1 and fibrillin 2. MFAP4 promotes coacervation of tropoelastin and has been localized to the elastin-microfibril interface [38]. In support of these findings, the addition of MFAP4 to dermal fibroblast cell culture enhances elastic fiber formation with a role in the assembly of microfibrils through a proposed interaction with fibrillin 1 [39]. Latent TGFβ binding protein 1 (187 kDa) interacts with fibrillin 1 [5], [40]. Of the three remaining differentially expressed genes, thrombospondin 2 (150–160 kDa) participates in skin collagen fibrillogenesis [41], while periostin (80–90 kDa) and tenascin C (250–300 kDa) are implicated in the pathogenesis of elastofibroma dorsi, a benign fibrous soft tissue disorder characterized by an excessive number of abnormal elastic fibers [42].

It may be that a number of these factors work together to enhance elastogenesis. To test this hypothesis, the older fibroblasts were cultured in FM and supplemented with CM that had been fractionated based on molecular weight. Fractions were divided into those containing factors <30 kDa, those between 30 and 100 kDa, and >100 kDa. Increased elastogenesis was obtained when the 30–100 kDa fraction was independently used to supplement the FM (Fig 3F) but did not reach the levels seen for the intact CM, which points to the involvement of multiple factors.

### 3.3 Enhanced elastogenesis with multiple tropoelastin treatments

**Fig. 4:**
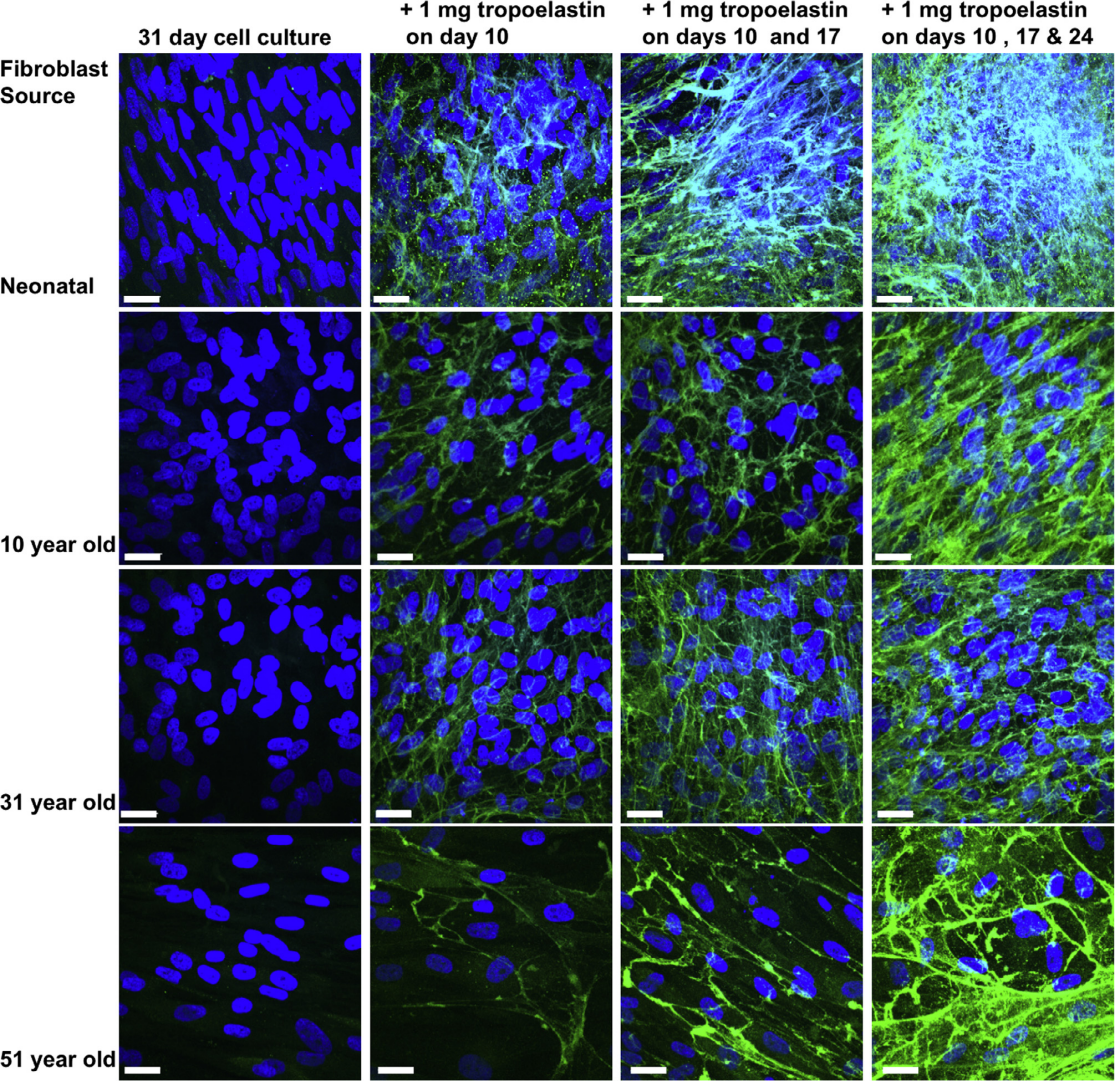
Enhanced elastogenesis through multiple tropoelastin treatments. Confocal images demonstrating increasing elastic network formation with repeated tropoelastin additions to cultured dermal fibroblasts sourced from different age groups (neonatal, 10, 31 and 51 year old). Elastic fibers were not evident in untreated cultures. Cultures were stained for elastin (green) and nuclei (blue). Scale bar = 20 μm. (For interpretation of the references to colour in this figure legend, the reader is referred to the web version of this article.)

**Fig. 5:**
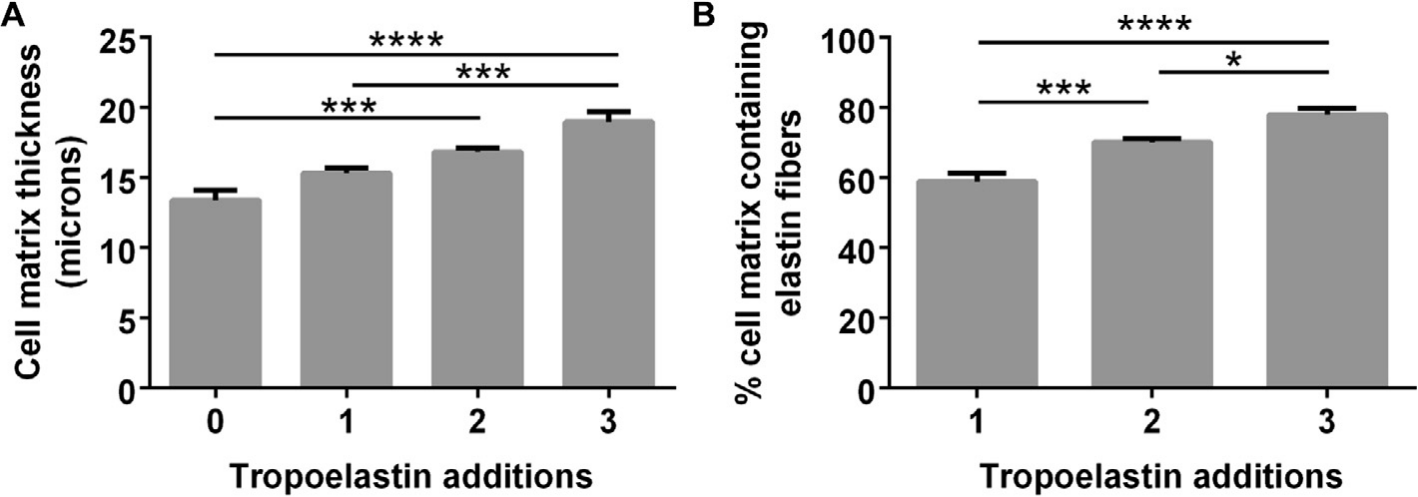
Effect of repeated tropoelastin addition on the cell matrix thickness (A) and elastin fiber content (B) of neonatal dermal fibroblasts cultured for 31 days. ^*^p < 0.05; ^**^p < 0.01; ^***^p < 0.001; ^****^p < 0.0001.

The elastogenic dependence by dermal fibroblasts on added tropoelastin was tested with multiple rounds of tropoelastin supplementation. An additional three tropoelastin treatments across a 31-day culture period demonstrated that fibroblasts from a range of age groups (0, 10, 31 and 51 year old donors) have the capacity to incorporate repeated doses of tropoelastin into a growing elastic network (Fig 4). Regardless of the number of tropoelastin additions (0–3) the total incubation time across all samples was 31 days. In the absence of exogenous tropoelastin supplementation there was no evidence of elastic fiber synthesis which demonstrates the requirement for added tropoelastin in fiber formation. This process was accompanied by increases in cell matrix thickness that correlated with each addition of the protein. Three treatments gave rise to a 1.5-fold increase in the thickness of a neonatal dermal fibroblast culture compared to one without tropoelastin supplementation (Fig 5A). Furthermore, the proportion of the cell matrix containing elastic fibers correspondingly increased from 59% with one tropoelastin treatment to 78% after three tropoelastin treatments (Fig 5B).

### 3.4 Elastic fiber enriched dermal substitute

A major cause of the deficiency in elastic fiber production is the failure to upregulate tropoelastin gene expression in postnatal tissues subject to injuries. Only low maintenance levels of tropoelastin mRNA are found in most elastic tissues in adults [43] which means that there is a chronic paucity of elastin in repairing full-thickness wounds.

**Fig. 6:**
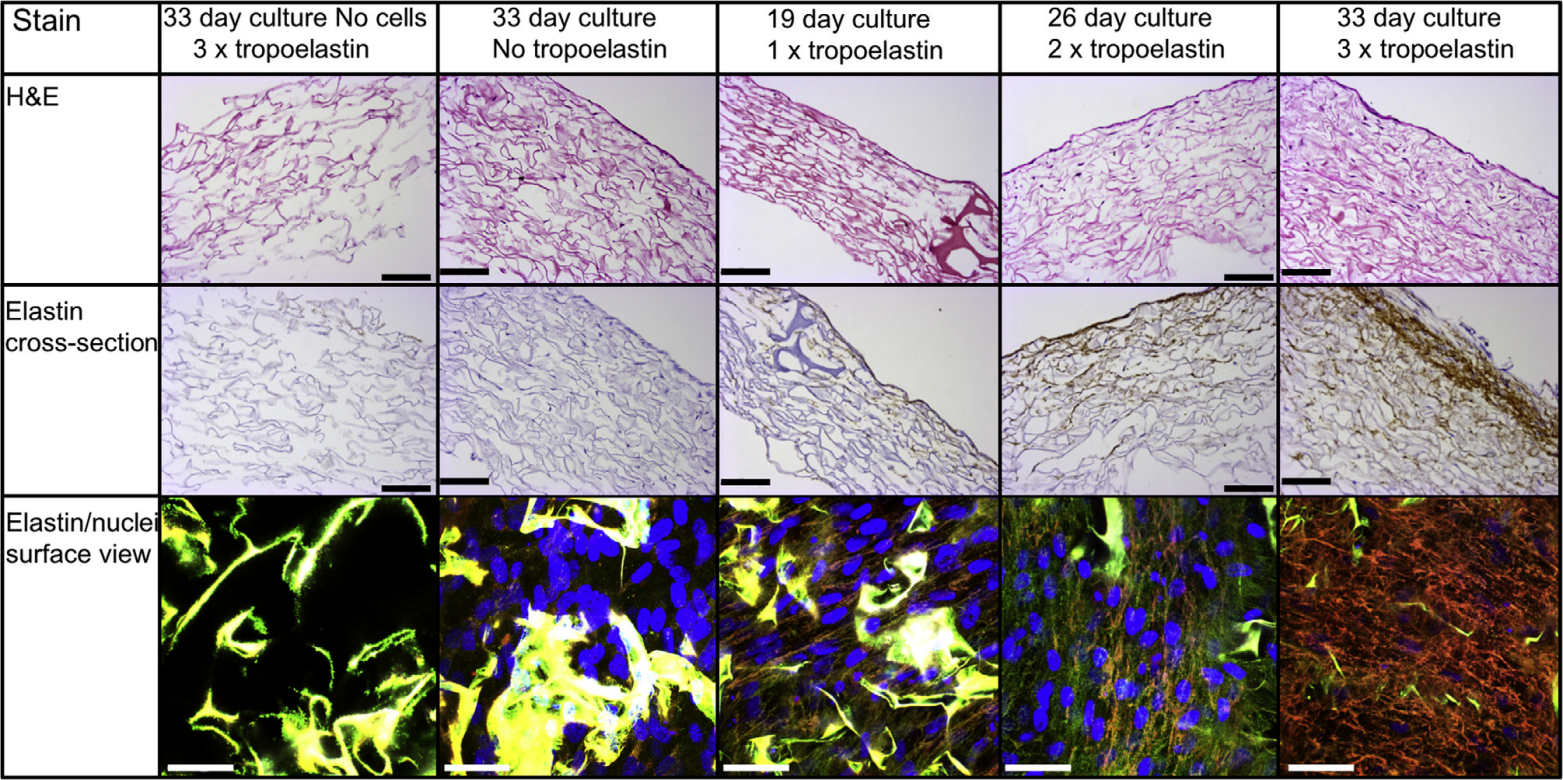
Elastin layered cell-containing dermal substitute. Bright field and confocal images showing the capacity for an extensive elastin network layer to be formed within a dermal substitute that is cultured with both dermal fibroblasts and repeated tropoelastin treatments. Control IDRT samples cultured with only tropoelastin or cells do not exhibit an elastin network layer. H&E cross-sections show fibroblast (purple nuclei) infiltration into the IDRT increases with time. DAB-based elastin stained cross-sections show the developing elastin layer (brown stain) on the upper surface of the dermal substitute. Confocal images of this surface layer reveal an extensive elastic fiber network (orange). To distinguish between the autofluorescing collagen matrix (yellow) and the elastin network, confocal images were produced by merging images obtained through excitation at 405 nm to detect DAPI stained nuclei (blue), 488 nm to detect elastin-stained FITC fluorescence and 559 nm to detect elastin autofluorescence. Maturing elastin fibers appear orange under these conditions. H&E and elastin cross-section images scale bar = 100 μm, confocal surface images scale bar = 50 μm. (For interpretation of the references to colour in this figure legend, the reader is referred to the web version of this article.)

We used the technology described here to circumvent this deficiency by pre-incubating donor fibroblasts with exogenous tropoelastin on IDRT, which is the leading commercial collagen-based dermal substitute. This approach delivered elastic fibers in the upper layer, which increased with the number of doses of tropoelastin (Fig 6). Elastin stained cross-sectional images confirmed the presence of elastin on the surface and within the scaffold. Only when cells utilize the supplemented tropoelastin do we see fibers that display both BA4 staining and intrinsic autofluorescence characteristic of elastin [21]. Elastic fibers were not evident in IDRT samples that were cultured with either cells and no tropoelastin or tropoelastin and no cells. Repeated applications of tropoelastin gave rise to a thick elastic fiber-containing layer at the top surface of the IDRT. Fluorescent elastin staining and confocal imaging confirmed the presence of an extensive network of elastin fibers in the upper layer of the IDRT, giving two effective layers: a lower IDRT region topped with a modified matrix enriched with patient elastic fibers.

**Fig. 7:**
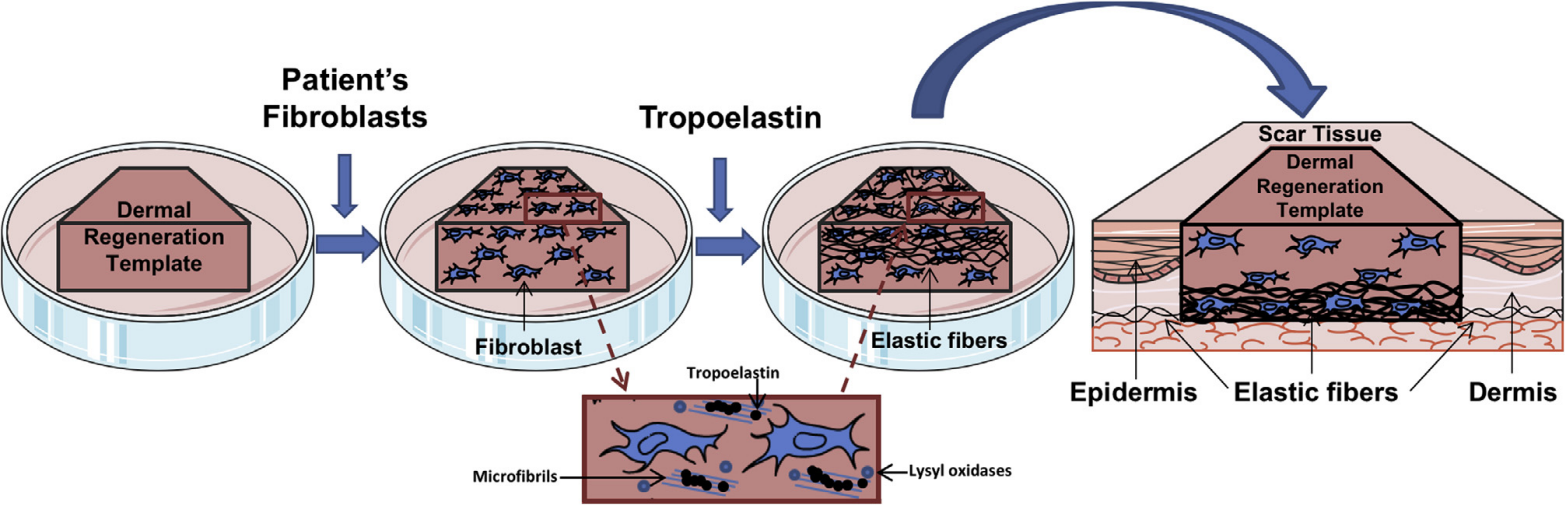
Proposed application for full thickness wound treatment. Patient dermal fibroblasts are cultured on a dermal regeneration template where they deposit elastic fiber proteins including microfibrillar proteins and lysyl oxidases. Treatment with repeated applications of tropoelastin leads to the formation of an extensive elastic fiber network on the upper surface of the template. After it has developed the cell-matrix can be inverted and positioned within a scar tissue site.

This design is attractive because it facilitates the delivery of a prefabricated elastic fiber network into the deep dermis during surgical treatment (Fig 7). This approach is appealing because this elastic fiber network is made using autologous dermal fibroblasts and therefore comprises autologous protein components. *In vivo* studies will assess its performance including whether this elastin net will persist and function. We have previously demonstrated that recombinant human tropoelastin is well tolerated [44]. This system is designed to be compatible with human clinical use, such as revision surgery, because of its emphasis on human donor cells and synthesized human extracellular matrix.

## 4 Conclusions

We describe a process and hybrid biomaterial intended to deliver tunable levels of histologically detectable patient elastin into full-thickness wound sites. This approach addresses a persistent unmet need because repairing wounds lack this elastic substratum. Previously, dogma asserted that elastin synthesis is attenuated in early childhood but we show here that we can overcome this restriction by adding exogenous tropoelastin, regardless of the age of the dermal fibroblast donor. We describe how to further enhance synthesis by older cells by using CM. This approach delivers elastin as a layer on the leading dermal repair template for contact with the deep dermis in order to deliver prefabricated elastic fibers to the physiologically appropriate site during surgery to repair scar tissue at sites of healing full thickness wounds.

## Funding

We acknowledge funding from the Australian Research Council, National Health and Medical Research Council and Wellcome Trust.

## Disclosures

ASW is the Scientific Founder of Elastagen Pty Ltd.

## Figures and Tables

**Table 1:** Genes differentially expressed in neonatal dermal fibroblasts compared to dermal fibroblasts sourced from a 51-year-old.

Gene Name	Gene Symbol	Signal Intensity 142BR cells (51 year old)	Signal Intensity NHF45C cells (neonatal)	p value	Fold Change
Fibrillin 2	FBN2	598 ± 37	4589 ± 733	0.0007	7.7
Fibulin 1	FBLN1	692 ± 66	3819 ± 204	<0.0001	5.5
Microfibrillar-associated protein 4	MFAP4	297 ± 47	1290 ± 74	<0.0001	4.3
Latent TGFβ binding protein 1	LTBP1	668 ± 36	2851 ± 291	0.0002	4.3
Thrombospondin 2	THBS2	1435 ± 173	5670 ± 1171	0.004	4.0
Periostin	POSTN	2154 ± 113	8115 ± 226	<0.0001	3.8
Tenascin C	TNC	1528 ± 247	4523 ± 694	0.002	3.0

## References

[1] Li D.Y., Brooke B., Davis E.C., Mecham R.P., Sorensen L.K., Boak B.B., Eichwald E., Keating M.T. (1998). Elastin is an essential determinant of arterial morphogenesis. Nature.

[2] Shapiro S.D., Endicott S.K., Province M.A., Pierce J., Campbell E.J. (1991). Marked longevity of human lung parenchymal elastic fibers deduced from prevalence of d-aspartate and nuclear weapons-related radiocarbon. J. Clin. Invest..

[3] Sivan S.S., Van El B., Merkher Y., Schmelzer C.E., Zuurmond A.M., Heinz A., Wachtel E., Varga P.P., Lazary A., Brayda-Bruno M., Maroudas A. (2012). Longevity of elastin in human intervertebral disc as probed by the racemization of aspartic acid. Biochim. Biophys. Acta.

[4] Kelleher C.M., McLean S.E., Mecham R.P. (2004). Vascular extracellular matrix and aortic development. Curr. Top. Dev. Biol..

[5] Raghunath M., Unsold C., Kubitscheck U., Bruckner-Tuderman L., Peters R., Meuli M. (1998). The cutaneous microfibrillar apparatus contains latent transforming growth factor-beta binding protein-1 (LTBP-1) and is a repository for latent TGF-beta1. J. Invest. Dermatol..

[6] Sherratt M.J. (2009). Tissue elasticity and the ageing elastic fibre. Age.

[7] Langton A.K., Sherratt M.J., Griffiths C.E., Watson R.E. (2010). A new wrinkle on old skin: the role of elastic fibres in skin ageing. Int. J. Cosmet. Sci..

[8] Zhang M., Pierce R.A., Wachi H., Mecham R.P., Parks W.C. (1999). An open reading frame element mediates posttranscriptional regulation of tropoelastin and responsiveness to transforming growth factor beta1. Mol. Cell. Biol..

[9] Rnjak J., Wise S.G., Mithieux S.M., Weiss A.S. (2011). Severe burn injuries and the role of elastin in the design of dermal substitutes. Tissue Eng. Part B Rev..

[10] Hirt-Burri N., Ramelet A.A., Raffoul W., de Buys Roessingh A., Scaletta C., Pioletti D., Applegate L.A. (2011). Biologicals and fetal cell therapy for wound and scar management. ISRN Dermatol..

[11] Hohlfeld J., de Buys Roessingh A., Hirt-Burri N., Chaubert P., Gerber S., Scaletta C., Hohlfeld P., Applegate L.A. (2005). Tissue engineered fetal skin constructs for paediatric burns. Lancet.

[12] Mithieux S.M., Weiss A.S. (2005). Elastin. Adv. Protein Chem..

[13] Wise S.G., Weiss A.S. (2009). Tropoelastin. Int. J. Biochem. Cell Biol..

[14] Wagenseil J.E., Mecham R.P. (2007). New insights into elastic fiber assembly. Birth Defects Res. C Embryo Today.

[15] Baldwin A.K., Cain S.A., Lennon R., Godwin A., Merry C.L., Kielty C.M. (2014). Epithelial-mesenchymal status influences how cells deposit fibrillin microfibrils. J. Cell Sci..

[16] Sabatier L., Djokic J., Hubmacher D., Dzafik D., Nelea V., Reinhardt D.P. (2014). Heparin/heparan sulfate controls fibrillin-1, -2 and -3 self-interactions in microfibril assembly. FEBS Lett..

[17] Papke C.L., Yanagisawa H. (2014). Fibulin-4 and fibulin-5 in elastogenesis and beyond: Insights from mouse and human studies. Matrix Biol..

[18] Hirai M., Ohbayashi T., Horiguchi M., Okawa K., Hagiwara A., Chien K.R., Kita T., Nakamura T. (2007). Fibulin-5/DANCE has an elastogenic organizer activity that is abrogated by proteolytic cleavage in vivo. J. Cell Biol..

[19] Wise S.G., Yeo G.C., Hiob M.A., Rnjak-Kovacina J., Kaplan D.L., Ng M.K., Weiss A.S. (2014). Tropoelastin: a versatile, bioactive assembly module. Acta Biomater..

[20] Yeo G.C., Baldock C., Tuukkanen A., Roessle M., Dyksterhuis L.B., Wise S.G., Matthews J., Mithieux S.M., Weiss A.S. (2012). Tropoelastin bridge region positions the cell-interactive C terminus and contributes to elastic fiber assembly. Proc. Natl. Acad. Sci. U.S.A.

[21] Yeo G.C., Baldock C., Wise S.G., Weiss A.S. (2014). A negatively charged residue stabilizes the tropoelastin N-terminal region for elastic fiber assembly. J. Biol. Chem..

[22] Dyksterhuis L.B., Carter E.A., Mithieux S.M., Weiss A.S. (2009). Tropoelastin as a thermodynamically unfolded premolten globule protein: the effect of trimethylamine N-oxide on structure and coacervation. Arch. Biochem. Biophys..

[23] Tu Y., Weiss A.S. (2010). Transient tropoelastin nanoparticles are early-stage intermediates in the coacervation of human tropoelastin whose aggregation is facilitated by heparan sulfate and heparin decasaccharides. Matrix Biol..

[24] Yeo G.C., Keeley F.W., Weiss A.S. (2011). Coacervation of tropoelastin. Adv. Colloid Interface Sci..

[25] Vrhovski B., Weiss A.S. (1998). Biochemistry of tropoelastin. Eur. J. Biochem..

[26] Martin S.L., Vrhovski B., Weiss A.S. (1995). Total synthesis and expression in *Escherichia coli* of a gene encoding human tropoelastin. Gene.

[27] Wu W.J., Vrhovski B., Weiss A.S. (1999). Glycosaminoglycans mediate the coacervation of human tropoelastin through dominant charge interactions involving lysine side chains. J. Biol. Chem..

[28] Rasband W.S. (1997–2016). ImageJ.

[29] Kozel B.A., Ciliberto C.H., Mecham R.P. (2004). Deposition of tropoelastin into the extracellular matrix requires a competent elastic fiber scaffold but not live cells. Matrix Biol..

[30] Wachi H., Sato F., Murata H., Nakazawa J., Starcher B.C., Seyama Y. (2005). Development of a new in vitro model of elastic fiber assembly in human pigmented epithelial cells. Clin. Biochem..

[31] Muiznieks L.D., Miao M., Sitarz E.E., Keeley F.W. (2016). Contribution of domain 30 of tropoelastin to elastic fiber formation and material elasticity. Biopolymers.

[32] Kozel B.A., Rongish B.J., Czirok A., Zach J., Little C.D., Davis E.C., Knutsen R.H., Wagenseil J.E., Levy M.A., Mecham R.P. (2006). Elastic fiber formation: a dynamic view of extracellular matrix assembly using timer reporters. J. Cell. Physiol..

[33] Czirok A., Zach J., Kozel B.A., Mecham R.P., Davis E.C., Rongish B.J. (2006). Elastic fiber macro-assembly is a hierarchical, cell motion-mediated process. J. Cell. Physiol..

[34] Zhang H., Hu W., Ramirez F. (1995). Developmental expression of fibrillin genes suggests heterogeneity of extracellular microfibrils. J. Cell Biol..

[35] Charbonneau N.L., Jordan C.D., Keene D.R., Lee-Arteaga S., Dietz H.C., Rifkin D.B., Ramirez F., Sakai L.Y. (2010). Microfibril structure masks fibrillin-2 in postnatal tissues. J. Biol. Chem..

[36] Kobayashi N., Kostka G., Garbe J.H., Keene D.R., Bachinger H.P., Hanisch F.G., Markova D., Tsuda T., Timpl R., Chu M.L., Sasaki T. (2007). A comparative analysis of the fibulin protein family. biochemical characterization, binding interactions, and tissue localization. J. Biol. Chem..

[37] Sasaki T., Gohring W., Miosge N., Abrams W.R., Rosenbloom J., Timpl R. (1999). Tropoelastin binding to fibulins, nidogen-2 and other extracellular matrix proteins. FEBS Lett..

[38] Pilecki B., Holm A.T., Schlosser A., Moeller J.B., Wohl A.P., Zuk A.V., Heumuller S.E., Wallis R., Moestrup S.K., Sengle G., Holmskov U., Sorensen G.L. (2016). Characterization of microfibrillar-associated protein 4 (MFAP4) as a tropoelastin- and fibrillin-binding protein involved in elastic fiber formation. J. Biol. Chem..

[39] Kasamatsu S., Hachiya A., Fujimura T., Sriwiriyanont P., Haketa K., Visscher M.O., Kitzmiller W.J., Bello A., Kitahara T., Kobinger G.P., Takema Y. (2011). Essential role of microfibrillar-associated protein 4 in human cutaneous homeostasis and in its photoprotection. Sci. Rep..

[40] Robertson I.B., Horiguchi M., Zilberberg L., Dabovic B., Hadjiolova K., Rifkin D.B. (2015). Latent TGF-beta-binding proteins. Matrix Biol..

[41] Calabro N.E., Kristofik N.J., Kyriakides T.R. (2014). Thrombospondin-2 and extracellular matrix assembly. Biochim. Biophys. Acta.

[42] Di Vito A., Scali E., Ferraro G., Mignogna C., Presta I., Camastra C., Donato G., Barni T. (2015). Elastofibroma dorsi: a histochemical and immunohistochemical study. Eur. J. Histochem..

[43] Dong X.R., Majesky M.W. (2012 Mar). Restoring elastin with microRNA-29. Arterioscler. Thromb. Vasc. Biol..

[44] Wang Y., Mithieux S.M., Kong Y., Wang X.Q., Chong C., Fathi A., Dehghani F., Panas E., Kemnitzer J., Daniels R., Kimble R.M., Maitz P.K., Li Z., Weiss A.S. (2015). Tropoelastin incorporation into a dermal regeneration template promotes wound angiogenesis. Adv. Healthcare Mater..

